# Influence of porosity on osteogenesis, bone growth and osteointegration in trabecular tantalum scaffolds fabricated by additive manufacturing

**DOI:** 10.3389/fbioe.2023.1117954

**Published:** 2023-01-27

**Authors:** Juyang Jiao, Qimin Hong, Dachen Zhang, Minqi Wang, Haozheng Tang, Jingzhou Yang, Xinhua Qu, Bing Yue

**Affiliations:** ^1^ Department of Bone and Joint Surgery, Department of Orthopedics, Renji Hospital, Shanghai Jiao Tong University School of Medicine, Shanghai, China; ^2^ Shenzhen Dazhou Medical Technology Co., Ltd., Shenzhen, Guangdong, China; ^3^ Center of Biomedical Materials 3D Printing, National Engineering Laboratory for Polymer Complex Structure Additive Manufacturing, Baoding, Hebei, China; ^4^ School of Mechanical and Automobile Engineering, Qingdao University of Technology, Qingdao, Shandong, China

**Keywords:** additive manufacturing, bone repair, osseointegration, porosity, tantalum scaffold, trabecular

## Abstract

Porous tantalum implants are a class of materials commonly used in clinical practice to repair bone defects. However, the cumbersome and problematic preparation procedure have limited their widespread application. Additive manufacturing has revolutionized the design and process of orthopedic implants, but the pore architecture feature of porous tantalum scaffolds prepared from additive materials for optimal osseointegration are unclear, particularly the influence of porosity. We prepared trabecular bone-mimicking tantalum scaffolds with three different porosities (60%, 70% and 80%) using the laser powder bed fusing technique to examine and compare the effects of adhesion, proliferation and osteogenic differentiation capacity of rat mesenchymal stem cells on the scaffolds *in vitro*. The *in vivo* bone ingrowth and osseointegration effects of each scaffold were analyzed in a rat femoral bone defect model. Three porous tantalum scaffolds were successfully prepared and characterized. *In vitro* studies showed that scaffolds with 70% and 80% porosity had a better ability to osteogenic proliferation and differentiation than scaffolds with 60% porosity. *In vivo* studies further confirmed that tantalum scaffolds with the 70% and 80% porosity had a better ability for bone ingrowh than the scaffold with 60% porosity. As for osseointegration, more bone was bound to the material in the scaffold with 70% porosity, suggesting that the 3D printed trabecular tantalum scaffold with 70% porosity could be the optimal choice for subsequent implant design, which we will further confirm in a large animal preclinical model for better clinical use.

## 1 Introduction

Bone tissue has a natural regenerative and self-healing capacity to repair minor injuries such as cracks. However, large bone defects caused by severe trauma, degenerative disease, congenital malformations or surgical removal of malignant tumors often require surgical intervention to reconstruct bone morphology and function so as to achieve complete healing (McDermott et al., 2019; Koons et al., 2020). Moreover, changes in the bone microenvironment caused by degenerative diseases, infections, osteoporosis, and bone metastases have a large influence on bone repair (Claes et al., 2012). Current bone repair materials successfully used in clinical settings are bioactive bone (homogeneous autologous bone/homogeneous allogeneous bone/xenogeneic bone), bioceramics (hydroxyapatite, calcium phosphate, tricalcium phosphate), inorganic/organic polymers (collagen, alginate, polylactic acid, polyethylene glycol) and biomedical metals (titanium, stainless steel and tantalum) (Webber et al., 2016; Gillman and Jayasuriya, 2021). Bioactive bone is the gold standard for bone repair materials due to its excellent osteogenic, osteoinductive and osteoconductive properties, but insufficient donor sources, collateral donor site damage, potential risk of infectious disease transmission and immunogenicity restrict its large-scale use (Amini et al., 2012). Bioceramics provide relatively high compression modulus and release bioactive ions, but are brittle (Jakus et al., 2016). Polymers are widely available and easy to modify, but also have weak mechanical properties and immunogenicity risks (Guo et al., 2021). Therefore, the above materials are mostly used to repair small, non-weight-bearing bone defects, while metal is the best solution for critical and weight-bearing bone defects due to its excellent mechanical properties and good biocompatibility (Pobloth et al., 2018). Pure titanium and titanium alloys (Ti6Al4V) are the most commonly used metal implants in clinical practice which have high mechanical strength, fatigue resistance and corrosion resistance, but they also suffer from aseptic loosening around the implant due to the stress-shielding effect caused by high elastic modulus, side effects related to corrosion-induced ion release, and poor osseointegration performance (Alipal et al., 2021).

Tantalum metal has excellent biological affinity, superior corrosion resistance, good mechanical ductility, bone formation and bone conduction properties, and is increasingly favored by clinicians and researchers (Levine et al., 2006). As an inert metal, tantalum can combine with oxygen to form a stable tantalum pentoxide (Ta_2_O_5_) passivation film, which is not easy to corrode; At the same time, the presence of oxide film is conducive to the formation of osteoid apatite coating and reduces the adhesion and colonization of bacteria (Han et al., 2019). Similar to the elastic modulus (110 GPa) of titanium alloys, the elastic modulus of dense bulk tantalum (186 GPa) is significantly higher than that of human cortical bone (3–30 GPa) and cancellous bone (0.02–2 GPa) (Wang et al., 2016; 2021). In addition, the high density (16.68 g/cm3) and high melting point (2,996°C) of tantalum constrain the industrial manufacturing and medical applications of tantalum materials (Black, 1994). It was not until the 1990s that a highly porous trabecular tantalum metal (Trabecular Metal^TM^(TM), Zimmer, Warsaw, IN, United States) prepared by chemical vapor deposition (CVD) was introduced and successfully used in clinical applications, including but not limited to femoral or tibial cone and augmentation in knee/hip arthroplasty revision, monoblock/modular tibial component, acetabular cup prosthesis, femoral necrosis reconstruction rods, interbody fusion cage, artificial shoulder prosthesis and dental implants (Bobyn et al., 1999; Christie, 2002; Cohen, 2002; Huang et al., 2021). However, there are still some intractable key problems with the CVD method. First, this traditional preparation technology is costly, time-consuming, and inefficient; second, it is difficult to prepare bone implants that are individually tailored by the patient to fit the shape of the anatomical site; and third, it is impossible to guarantee the accuracy of the design and control of the porous structural features of the scaffold.

Additive manufacturing, as an advanced, powerful and mature processing technology, overcomes the deficiencies of traditional techniques and can be used to manufacture porous metal scaffolds with complex layered structures and high precision, which has very attractive application prospects (Bose et al., 2018). Additive manufacturing technologies are still progressing rapidly, including but not limited to selective laser melting (SLM), electron beam melting (EBM), selective laser sintering (SLS), laser engineered net shaping (LENS), fused deposition modeling, binder jetting, and direct metal printing (Chen Y. et al., 2020). SLM (Wauthle et al., 2015; Guo et al., 2019; Wang et al., 2019; Yang et al., 2020) and LENS (Balla et al., 2010; Bandyopadhyay et al., 2019) has been successfully used in the preparation of porous tantalum scaffolds. Among them, SLM, in particular Laser-based powder bed fusion (LPBF), stands out for its good stability, high efficiency, smooth surface finish and the ability to precisely tune the internal pore architecture of the porous structure.

The porous structure is critical for the mechanical and biological properties of the implant. Porosity, pore geometry, pore size, strut diameter and interconnectivity of pores are important parameters for the topology design of porous architectures (Gao et al., 2021). Among these, porosity is regarded as the dominant effect affecting the mechanical (stiffness and hardness) and biological properties of the implant, with other parameters such as pore geometry being a non-negligible secondary effect (Al-Ketan et al., 2018; Kelly et al., 2018). On the one hand, high porosity reduces the mechanical strength of metal scaffolds and subsequently achieves an elastic modulus comparable to that of bone, which helps to reduce stress shielding; on the other hand, high porosity provides a large specific surface area to promote cell migration as well as nutrient delivery and improves osseointegration. Several studies have reported the effect of porosity on the performance of porous metal scaffolds (Cheng et al., 2014; Chen Z. et al., 2020; Pei et al., 2021). Cheng et al. prepared a titanium alloy scaffold mimicking the structure of human cancellous bone using SLS and compared the effects of three porosities (16%, 38% and 70%, respectively) on *in vitro* osteogenesis, and found that the scaffold with 70% porosity was more favorable for osteoblast proliferation and differentiation (Cheng et al., 2014). Chen et al. compared Ti6Al4V ELI scaffolds with 60% and 70% porosity and showed that the scaffold with 60% porosity had the best bone growth outcome (Chen Z. et al., 2020). Among the porous tantalums prepared by additive materials, Wauthle et al. prepared highly porous tantalum implants (80% porosity) using SLM and demonstrated their excellent osteoconductivity and mechanical properties *in vitro* and *in vivo* (Wauthle et al., 2015). The above contradictory results may be related to the pore structure, material properties, preparation process, etc. Therefore, further studies are needed to comprehensively adjust the porous structure characteristics to achieve the optimization of mechanical and biological properties. Human cancellous bone is a complex morphologically irregular porous structure with porosity ranging from 50% to 90% and pore size of 300–500 μm, with non-homogeneous anisotropic properties (Li et al., 2017). The only porous tantalum implant successfully used in clinical practice to date, Trabecular Metal, uses a bone trabecular structure with 70%–85% porosity and an average pore size of 400–600 μm, a unique biomimetic structure that seems to have even more outstanding advantages (Wang et al., 2020). Yang et al. have successfully prepared porous tantalum scaffolds with a more refined trabecular bone mimetic structure using LPBF previously. Compared with the CVD-prepared TM, the trabecular bone tantalum scaffold prepared by SLM has the same porosity, interconnectivity of pores as well as larger pores and coarser filament diameter, and comparable mechanical properties to human cancellous bone (Yang et al., 2020). However, the biological properties of SLM-prepared trabecular tantalum scaffolds have not been fully investigated, especially the effect of porosity. In order to improve the reliability of implants in medical applications and for better clinical translation, there is an urgent need to systematically investigate the effects of porosity on bone formation, bone ingrowth and osseointegration of SLM-prepared trabecular tantalum scaffolds.

Therefore, the aim of this study was to explore the optimal porosity of SLM-prepared trabecular tantalum scaffolds for bone ongrowth and bone ingrowth. To this end, a series of trabecular bone tantalum scaffolds with different porosity were prepared by LPBF and characterized, followed by an *in vitro* study of their cytocompatibility and osteogenic ability, and finally a comparison of bone growth ability and biosafety in a rat femoral bone defect model.

## 2 Methods

### 2.1 Material preparation and characterization

We designed three types of porous tantalum scaffolds (60%, 70% and 80%) with different porosity of bionic trabeculae (denoted as Ta T60, Ta T70 and Ta T80, respectively) and prepared porous tantalum discs (10 mm in diameter × 3 mm in height) for *in vitro* studies and porous tantalum cylinders (3 mm in diameter × 5 mm in height) for *in vivo* studies with the above structures by 3D printing technology. Specifically, the bionic bone trabeculae structure and 3D conformation of the porous tantalum scaffold were designed by Rhino3D NURBS V7.0 (Robert McNeel & Assoc., Seattle, WA, United States) and Materialise Magics V22.0 (Materialise N.V., Leuven, Belgium). Based on the aforementioned computer-aided design (CAD) model, The sample was prepared by Dazhou Medical Co., Ltd. (Shenzhen, Guangdong, China)] ulitizing PBLF additive manufacturing system Farsoon FM271M (Farsoon Technologies Co., Ltd., Changsha, Hunan, China). TEKMATTM Ta-45 powder (TEKNA Advanced Materials Inc., Sherbrooke, QC, Canada) was used, and the powder was melted by the laser under reasonable laser parameter regulation, stacked layer by layer, and solidified into shape. The final sample is obtained after sandblasting and annealing treatment. Finally, sufficient ultrasonic shaking and cleaning were applied to remove the unfused particles from the support.

The pore characteristics, surface morphology and elemental composition of the samples were determined using field emission scanning electron microscopy (FE-SEM, Hitachi S4800, Japan), energy disperse spectroscopy (EDS) and ImageJ were used for the analysis. The actual porosity of the samples was calculated using the weight method at standard atmospheric pressure according to the following formula.

$$Porosity\;\left(\%\right)=\frac{sample\;volume\times material\;density-actual\;sample\;weight}{sample\;volume\times material\;density}\times 100\%$$ (1)

Static mechanical testing of porous tantalum scaffolds with different porosity including compression, bending and torsion experiments have been reported in previous studies (Yang et al., 2020).

### 2.2 *In vitro* cytocompatibility assessment

#### 2.2.1 Culture of cells on materials

All materials were sonicated and vortexed for 2 h and washed with ultraclean water to remove unmelted powder prior to use. After autoclaving and drying, porous tantalum scaffolds were placed in 48-well plates with a small amount of Minimum Essential Medium-alpha (α-MEM, Hyclone) for infiltration. Rat bone marrow mesenchymal stem cells (rBMSCs) were extracted and cultured as previously described (Huo et al., 2021). The cell harvesting was approved by the Animal Ethics Committee of Renji Hospital, Shanghai Jiao Tong University School of Medicine. Briefly, cells were cultured in α-MEM containing 1% penecillin-streptomycin and 10% fetal bovine serum (FBS, Hyclone, Logan, UT, United States) in an incubator (37°C, 5% CO2 and 95% air) and maintained in an incubator with fluid exchange every 3 days. Cells passed to the second or third generation were used for subsequent experiments. If not otherwise specified, rBMSCs were inoculated on the surface of the scaffolds at a density of 2 × 10^4^ cells/well in 48-well plates with α-MEM submerged scaffolds and fluid exchanges every other day.

#### 2.2.2 Cytotoxicity and hemolytic reactions

Cytotoxicity and proliferation on the scaffold were assessed by Cell Counting Kit-8 (CCK-8) assay and hemolysis assay. The group inoculated with cells alone without scaffold served as control. Cells on scaffolds were assayed on days 1, 3, and 5 using CCK-8 reagent (Dojindo, Kumamoto, Japan). The original medium was replaced with fresh complete medium containing 10% CCK-8 and incubated in the incubator for 2 h. After incubation, 100 μL of supernatant per well was added to a new 96-well plate, and the optical density (OD) values at 450 nm were measured using a microplate reader (BioTek microplate reader). Each experiment was repeated three times. In addition, fresh blood samples were taken from the tail vein of rats for the hemolysis test, and the blood cells were collected by centrifugation at 3,000 rpm for 5 min. Phosphate buffer solution (PBS) was washed three times and resuspended to reach a final erythrocyte concentration of 4% (v/v). The sample was placed in a centrifuge tube, and a sufficient amount of fresh erythrocyte suspension was added. After 2 h incubation at 37°C, samples were centrifuged at 3,000 rpm for 3 min. 100 μL of supernatant was transferred from each tube to a 96-well plate, and the OD value was detected at 560 nm. The 0.1% Triton X-100 solution and PBS were used as positive control and negative control, respectively, and three parallel groups of each sample were used. The hemolysis rate (%) was calculated according to the following formula:

$$Hemolysis\;rate\left(\%\right)=\frac{OD560\left(Sample\right)-OD560\left(PBS\right)}{OD560\left(Triton\right)-OD560\left(PBS\right)}\times 100\%$$ (2)

(OD560 (Sample), OD560 (PBS) and OD560 (Triton) are the OD value of samples, PBS and Triton X-100 solution at 560 nm, respectively).

#### 2.2.3 Live/dead cell staining

rBMSCs (4×10^4^ cells/well) on the different scaffold were cultured for 7 days, and cell viability was assessed using Calcein/PI Live/Dead Assay Kit (#C2015M, Beyotime Biotechnology, Shanghai) according to the instructions. Briefly, cells on the samples were added to the Calcein/PI working solution, incubated for 30 min at 37°C in the dark and the staining effect was observed under the confocal laser scanning microscope (CLSM).

#### 2.2.4 SEM observation of cell morphology

Cells were cultured as above for 1 day (density of rBMSCs was 2×10^4^ cells/well), and the cells on the surface of the scaffolds were washed 3 times with PBS and fixed overnight at 4°C in 2.5% glutaraldehyde solution. Then, the cells were washed 3 times with deionized water, dehydrated in alcohol solution with stepwise concentrations (30%, 50%, 70%, 80%, 90%, and 100%) for 10 min, dried and sprayed with gold, and observed under a scanning electron microscope (Carl Zeiss, Germany).

### 2.3 *In vitro* osteogenic response assessment

#### 2.3.1 Cell culture

rBMSCs were seeded at a density of 2 × 10^4^ cells/well on the surface of the material as described previously, and after 24 h the complete medium was replaced with an osteogenic induction solution (containing 10% FBS, 1% penecillin-streptomycin, 100 nmol/L dexamethasone, 10 mmol/L β-glycerolphosphate and 50 mmol/L ascorbic acid) and continue to incubate at 37°C in a humidified incubator with 5% CO2, followed by fluid exchanges at 2-day intervals.

#### 2.3.2 Alkaline phosphatase (ALP) staining and activity quantification

ALP staining and activity quantification of cells on the material surface were performed on days 7 and 14. Cells on the surface of the material were fixed with 4% paraformaldehyde for 1 min and stained by BCIP/NBT Alkaline Phosphatase Color Development Kit (#C3206, Beyotime Biotechnology, Shanghai) according to the instructions. Incubated for 30 min at room temperature in the dark, washed and dried, and took pictures. Meanwhile, ALP activity was assayed using the Alkaline Phosphatase Assay Kit (#P0321M, Beyotime Biotechnology, Shanghai). In brief, cells on the scaffold were washed with PBS, lysed on ice for 10 min with 0.1% Triton X-100, and the supernatant was removed. The absorbance was measured at 405 nm according to the operating instructions. In addition, total cellular protein of cells on the surface of each material was quantified using the BCA Protein Assay Kit (#23227, Thermo Scientific Pierce, Rockford, United States), and absorbance was measured at 562 nm. The ALP/total protein ratio was calculated to quantify ALP activity.

#### 2.3.3 Alizarin red S staining and semi-quantification

Cells on scaffolds were examined after 14 and 21 days of culture using the Alizarin Red S Staining Kit for Osteogenesis (#C0138, Beyotime Biotechnology, Shanghai). Briefly, cells on scaffolds were fixed with 4% paraformaldehyde for 20 min, washed 3 times with PBS, stained with Alizarin Red S staining solution at room temperature for 30 min, washed with deionized water and photographed for mineralized nodules. After that, 10% cetylpyridinium chloride was added to lyse the mineralized nodules and the absorbance was measured at 620 nm for semi-quantitative analysis.

#### 2.3.4 Quantitative real-time fluorescent PCR (qRT-PCR)

rBMSCs were inoculated at a density of 5 × 10^4^ cells/well on the material surface in 24-well plates for 7 and 14 days. Total RNA was extracted from rBMSCs using Simply P Total RNA Extraction Kit (#BSC52M1, Bioer Technology, Hangzhou, China). Next, reverse transcription reactions of total RNA were performed with the Primierscript RT Master kit (as indicated) to obtain cDNA. Osteogenic-related genes including ALP, bone morphogenetic protein 2 (BMP2), C-X-C motif chemokine ligand 12 (CXCL12), Runt-related transcription factor 2 (RUNX2), and osteocalcin (OCN) were analyzed by qRT-PCR using a TB Green Premix Ex TaqII (Takara, Japan) on a fluorescent quantitative PCR instrument (QuantStudio 7, Thermo Scientific, United States) was performed. The relative expression of the above genes was calculated using the internal reference gene GAPDH as a control. The primers were synthesized by Sangon Biotech (Shanghai, China), and the sequences are shown in Table 1.

**TABLE 1:** Primer sequences of rBMSCs used for qRT-PCR in this study.

Target gene	Direction	Primer sequence (5' to 3')
ALP	Forward	TCG CCT ATC AGC TAA TGC AC
	Reverse	GCC TTC TCA TCC AGT TCA TAT TCC
BMP-2	Forward	AGC ATG TTT GGC CTG AAG CAG AGA
	Reverse	TGA AAG TTC CTC GAT GGC TTC
CXCL-12	Forward	CCG ATT CTT TGA GAG CCA TGT
	Reverse	CAG ACT TGT CTG TTG TTG CTT
OCN	Forward	TAT GGC ACC ACC GTT TAG GG
	Reverse	CTG TGC CGT CCA TAC TTT CG
RUNX-2	Forward	CAA ACA ACC ACA GAA CCA CAA G
	Reverse	CTC AGA GCA CTC ACT GAC TC

### 2.4 *In vivo* bone ingrowth and biosafety assessment

#### 2.4.1 Surgical procedures

All animal experiments and operations were approved by the Animal Ethics Committee of Renji Hospital, Shanghai Jiao Tong University School of Medicine. We established a rat model of bone defect repair in the lateral femoral condyle. A total of 36 male SD rats (12 weeks old, mean weight 350 g ± 25.2 g) were obtained from the Shanghai Lab. Animal Research Center and randomly divided into three groups: 1) porous tantalum scaffold with 60% porosity (denoted as Ta T60); 2) porous tantalum scaffold with 70% porosity (denoted as Ta T70), and 3) porous tantalum scaffold group with 80% porosity (denoted as Ta T80). Each rat was anesthetized with 2% pentobarbital sodium (0.2 mL/100 g, intraperitoneal injection). After shaving and disinfection of the right lower extremity of the rats, a 3-mm diameter, 5-mm deep bone defect perpendicular to the bone surface was made. After saline rinsing to remove the bone debris, porous tantalum scaffolds with different porosity were implanted. Finally, the wound was flushed with saline and sutured layer by layer. Six and 12 weeks after implantation, the rats (6 per group) were euthanized to collect femoral samples. At 4 and 2 weeks before rats were sacrificed, alizarin red (30 mg/kg) and calcein (20 mg/kg) were injected intraperitoneally to mark the new bone formation process.

#### 2.4.2 Imaging assessment of the osteogenic properties of porous tantalum scaffolds *in vivo*

At 6 and 12 weeks postoperatively, intact right femurs of rats were obtained and fixed in 4% paraformaldehyde. The imaging characteristics of the porous tantalum scaffold were routinely evaluated using X-ray and micro-CT. X-ray frontal and lateral radiographs of the intact femur were taken using an X-ray imaging system (M-20, Faxitron, United States). Meanwhile, the femoral condyles and femur were scanned using micro-CT (SkyScan1076, Bruker, Belgium) to detect bone growth.

#### 2.4.3 Histological and histometric analysis of bone ingrowth in porous tantalum scaffolds

After completion of imaging, rat femoral specimens were subjected to hard tissue sectioning stained with Van Gieson (VG) and methylene blue (MB). The stained sections were imaged by a high-resolution microscope and an automated digital section scanner. Images of bone ingrowth within the scaffold with different magnifications were obtained. Semi-quantitative analysis of bone ingrowth was performed by Image Pro Plus 6.0 software (Media Cybernetic, Rockville, MD, United States). Two methods were used to analyze bone ingrowth and osseointegration: one was to calculate the relative bone area (RBA), which is the area of new bone divided by the available void area (available void area = total area—metal area), and the other was to calculate the bone implant contact (BIC) index, which is the length of direct contact with bone at the interface/total length of the interface. Fluorescent labeling of transverse sections was observed with a fluorescence microscope (Leica, Germany). The excitation/emission wavelengths of alizarin red and calcein were 543/580–670 and 488/500–550 nm, respectively. Other unstained hard tissue sections were gold sprayed and the morphological and compositional changes of bone and scaffold were characterized by SEM and EDS.

#### 2.4.4 *In vivo* biosafety assessment of porous tantalum scaffolds

The general condition of the rats including body temperature, weight change, and wound healing were observed daily after surgery. At 12 weeks postoperatively, arterial blood was randomly drawn from rats in each experimental group by cardiac blood collection (n = 3). Routine blood and blood biochemical parameters, including alanine aminotransferase (ALT), blood urea nitrogen (BUN) and creatine kinase (CK), were measured and compared. Meanwhile, heart, liver, spleen, lung and kidney organ specimens were obtained from each experimental group and HE staining was performed to assess the possible organ pathological damage.

### 2.5 Data analysis

Data analysis was performed using SPSS 26.0 statistical software (SPSS Inc., Chicago, United States). Numerical data are reported as mean ± standard deviation (SD). Statistical differences were analyzed by unpaired two-tailed Student's t-test or one-way ANOVA with Tukey *post hoc* comparisons. *p* < 0.05 were considered to be statistically significant.

## 3 Results

### 3.1 Characterization of porous tantalum scaffolds

**FIGURE 1:**
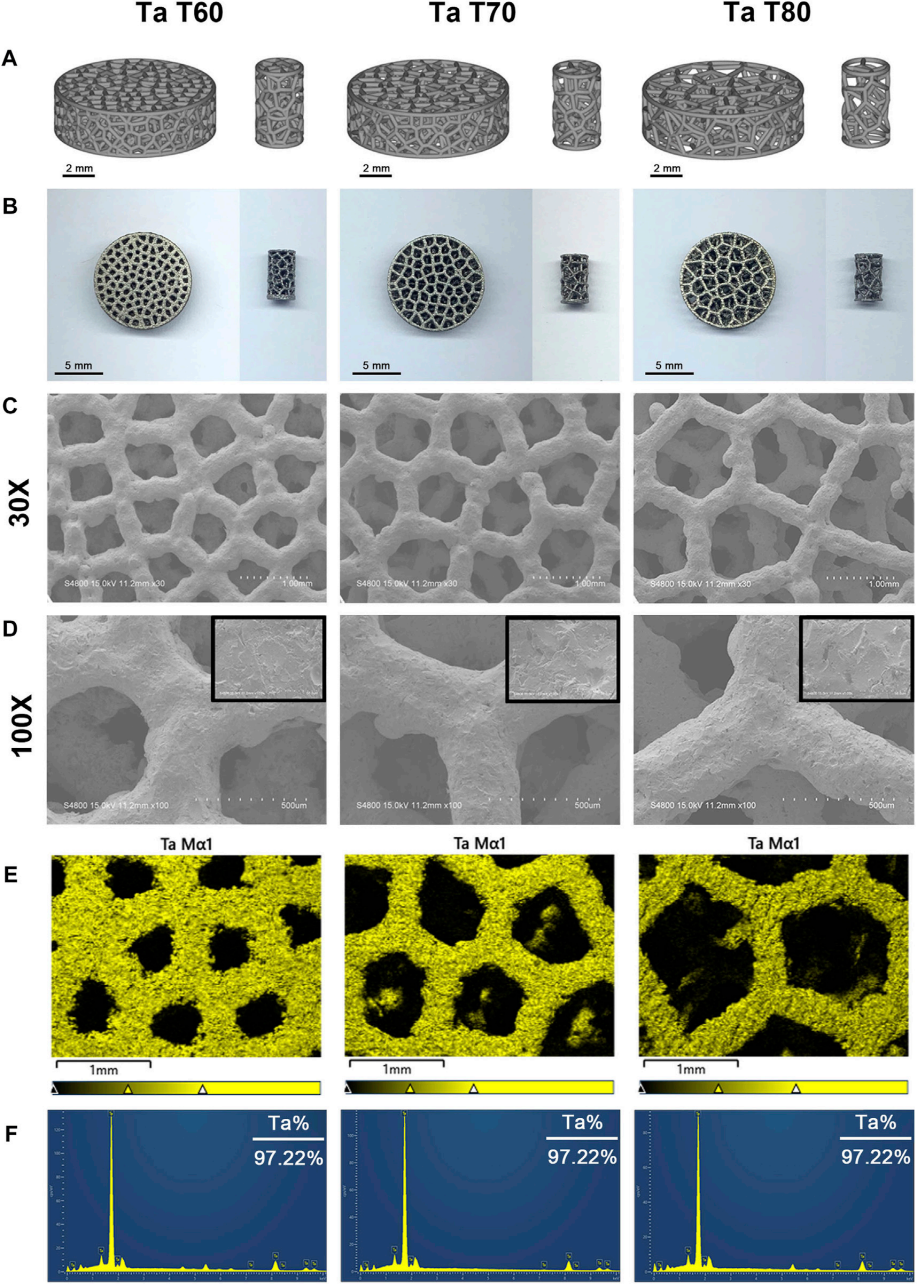
Material characterization of three porous tantalum scaffolds with different porosities. 3D modeling **(A)** and general appearance **(B)** of porous tantalum scaffolds (left: disks, right: cylinders). **(C)** and **(D)** Surface morphology of porous tantalum scaffolds under electron microscopy at ×30 **(C)** and ×100 **(D)** magnification, respectively (The upper right corner of Figure D is 1,000x magnification). EDS mapping **(E)** and spectra **(F)** show the elemental distribution and composition of each scaffold.

Figure 1A show the modeling and general appearance of porous tantalum scaffolds (discs and cylinders) with different porosities. Macroscopically, the surface of the scaffolds is smooth and flat, the pore structure is trabecular bone mimetic, and the pore size of each scaffold varies, which is consistent with the model. Then, we used SEM to further observe the microstructure of each scaffold (Figure 1C). The results showed that the strut diameter of each scaffold was uniform and complete, with the similar micro/nano rough surface, almost no unfused particles were present, and the pore size was consistent with the macroscopic one. In addition, the EDS results proved that all three porous tantalum scaffolds have only the presence of tantalum and oxygen elements, and the percentage of tantalum elements is basically the same (Figure 1D). The above results confirmed that pure tantalum porous scaffolds were successfully prepared by SLM with different pore sizes. Further, we compared the structural parameters such as porosity of each scaffold, and the structural parameters as well as mechanical properties of the three porous tantalum scaffolds are summarized in Table 2.

**TABLE 2:** Structural parameters and mechanical properties of 3D-printed porous tantalum scaffolds with different porosities.

			Groups		
			Ta T60	Ta T70	Ta T80
Structural parameters	Porosity (%)	D	60	70	80
		A	56.4	66.7	79.3
	Average pore diameter (μm)	D	450	600	800
	Strut diameter (μm)	D	300	300	300
		A	316.8 ± 17.8	320.3 ± 15.4	330.1 ± 15.4
	Pore interconectivity (%)	D	100	100	100
		A	99.99	99.99	99.99
Mechanical properties Yang et al. (2020)	Compressive strength (MPa)		59.5 ± 0.2	33.2 ± 0.4	14.2 ± 1
	Compressive modulus (GPa)		3.3 ± 0.3	3 ± 0.2	1.5 ± 0.4
	Bending strength (GPa)		97 ± 4.2	52.8 ± 2.6	23 ± 0.8
	Shear modulus (GPa)		6.8 ± 0.3	2.8 ± 0.6	1.2 ± 0.2
	Torsion strength (MPa)		41.2 ± 0.8	28 ± 0.3	11.2 ± 0.6

Values are represented as the mean ± SD (n = 5). A, actual; D, designed.

### 3.2 *In vitro* cytocompatibility

**FIGURE 2:**
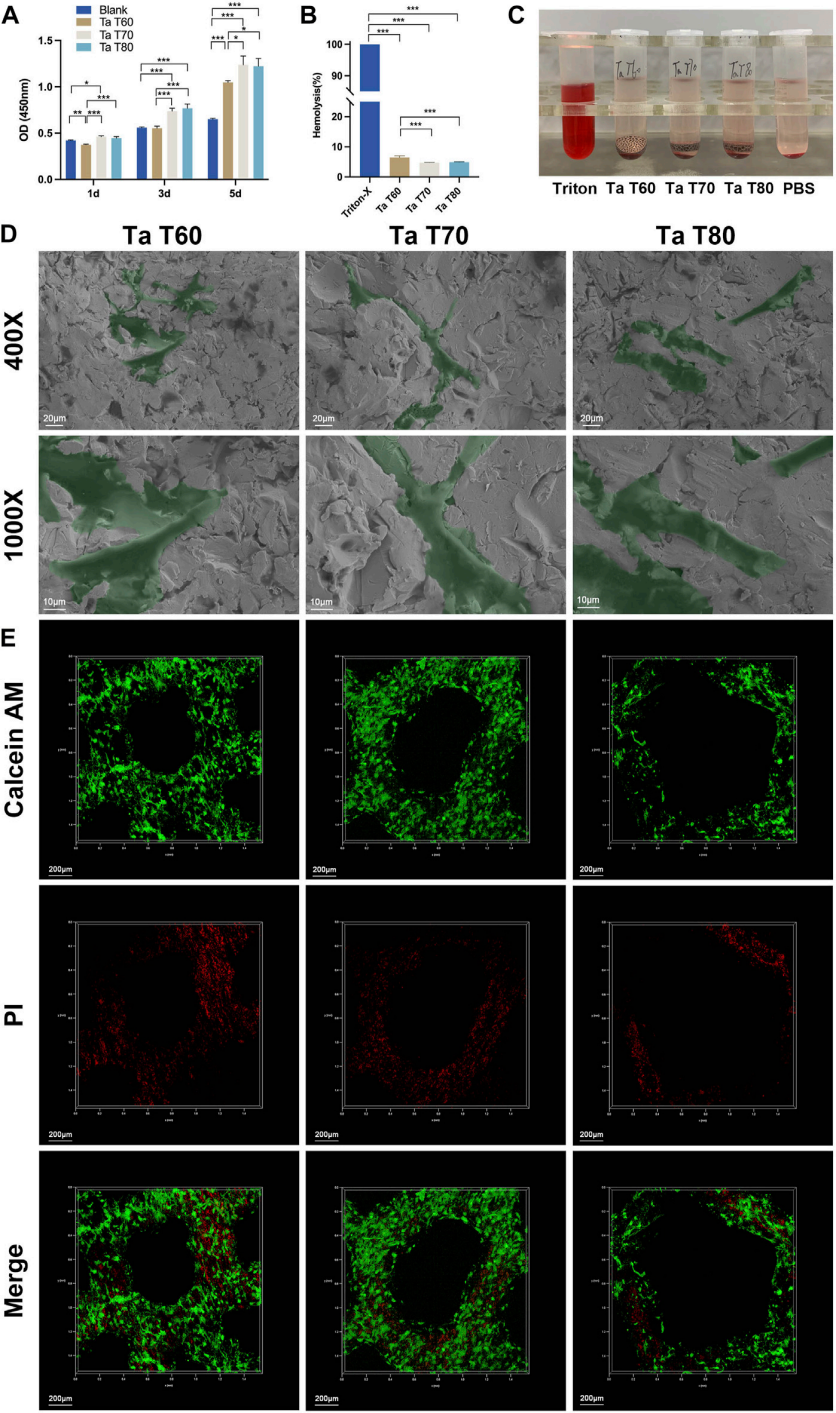
*In vitro* cytocompatibility of porous tantalum scaffolds and their effects on the growth of rBMSCs. **(A)** Activity of rBMSCs cultured on each tantalum scaffold for 1, 3 and 5 days. Quantitative analysis **(B)** and qualitative analysis **(C)** of hemolysis of blood cells cultured with tantalum scaffolds. **(D)** Representative SEM images of rBMSCs grown in the surface of tantalum scaffolds for 1 day. **(E)** Live/dead cell staining for rBMSCs cultured on tantalum scaffolds for 7 d. Data are represented as mean ± standard deviation (SD). (n = 3, *p < 0.05; **p < 0.01; ***p < 0.001.

First, we assayed CCK-8 after culture of rBMSCs on tantalum scaffolds for 1, 3 and 5 days to assess cytotoxicity. As shown in Figure 2A, there was no significant difference between the cells on the three scaffolds and the blank control at day 1. On days 3 and 5, cells on the materials were statistically higher in each group than in the blank group (except for the Ta T60 group in day 3). The materials showed an increasing trend at all three time points, and comparison between groups showed that cells in the Ta T70 and Ta T80 group were higher than that of Ta T60 group. The results showed that all three porous tantalum scaffolds with different porosity were non-cytotoxic and promoted the proliferation of rBMSCs. Next, we used hemolysis assay to assess the compatibility of the material and blood cells. Qualitative results (Figure 2B) showed that all tubes with the scaffold were clear and bright compared to the positive control. The quantitative results (Figure 2C) suggested that the hemolysis rates of Ta T60, Ta T70 and Ta T80 were 6.46%, 4.74% and 4.95%, respectively, which were within the normal range, indicating that there was no hemolytic side effect of the materials. We also performed live/dead cell staining on cells cultured on the materials for 7 days to assess the cell state. The CLSM results (Figure 2D) showed that most cells on all scaffolds were green fluorescent, and the green fluorescence intensity was in the order of Ta T60, Ta T70 and Ta T80 from highest to lowest (Supplementary Figure S1). Interestingly, the green fluorescence signal near the nodes on the surface of all materials was stronger than that on the struts. However, the green/red fluorescence signal of Ta T60 group was slightly lower than that of Ta T70 and Ta T80, but not statistically different. In addition, we observed the morphology of rBMSCs cultured on the material surface for 1 day by SEM, and as shown in Figure 2E, the cells on all materials were well spread, with flat morphology and extended multiple pseudopods. The above illustrates the superior cytocompatibility of the 3D-printed porous tantalum scaffold.

### 3.3 *In vitro* osteogenic properties

**FIGURE 3:**
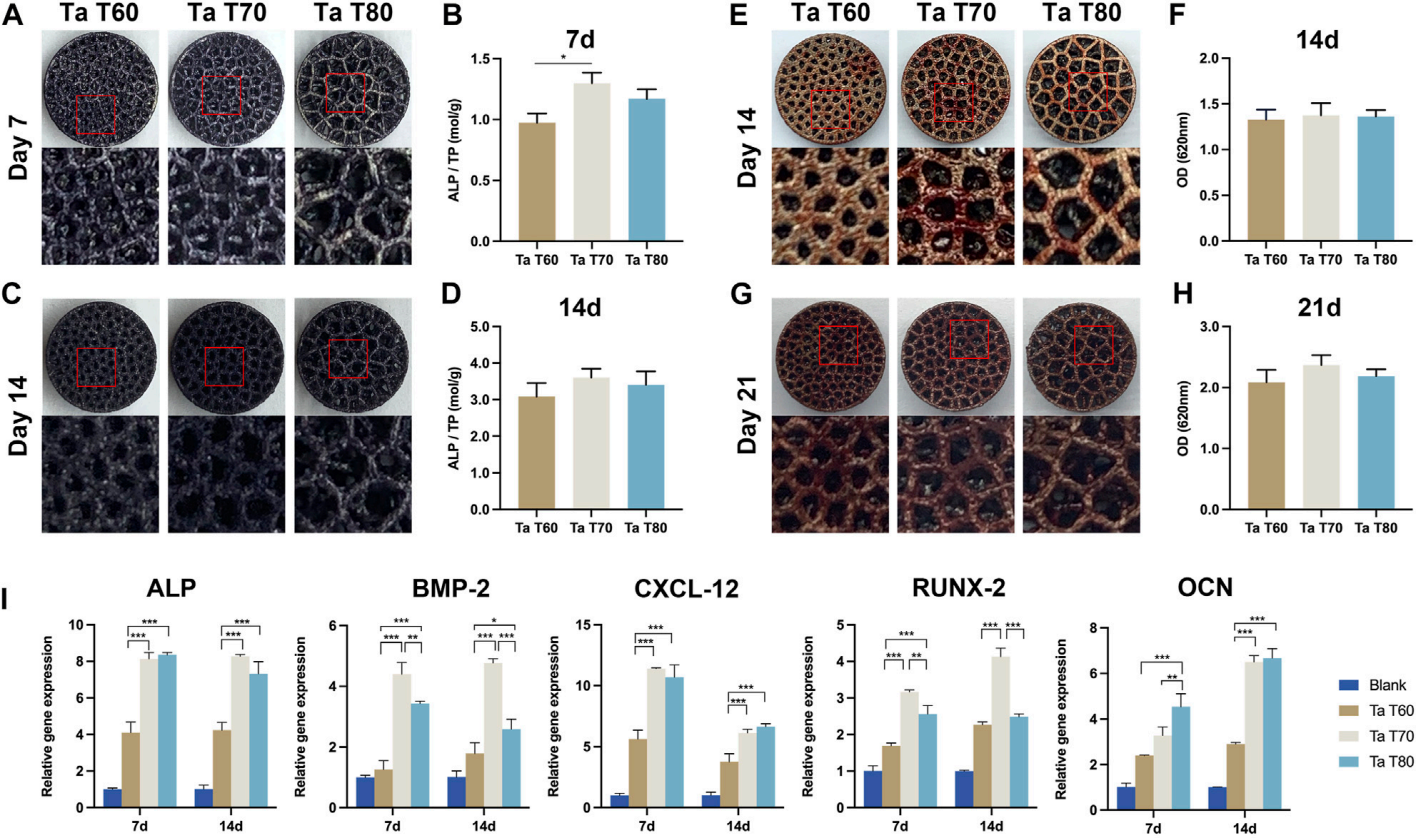
*In vitro* osteogenic effect of rBMSCs in each porous tantalum scaffold. **(A)** and **(C)** Appearance of ALP staining at 7d and 14d. **(B)** and **(D)** Quantitative assessment of ALP activity at 7d and 14d of culture. **(E)** and **(G)** Appearance of alizarin red staining at 14d and 21d. **(F)** and **(H)** Semi-quantitative analysis of alizarin red staining. **(I)** Osteogenesis-related gene expression at 7d and 14d of culture. Data represent mean ± SD. (n = 3, *p < 0.05; **p < 0.01; ***p < 0.001).

We performed qualitative and quantitative assays of ALP and calcium deposition by ALP staining and ARS staining, respectively, to assess the early and late osteogenic differentiation potential of porous tantalum scaffolds with different porosity. Figure 3A reflects the ALP assay on each group of material at 7 and 14 days, and the results showed that the amount of ALP was slightly higher in the Ta T60 group than in the Ta T70 and Ta T80 groups at 7 days, and the amount of ALP was significantly higher in all three groups at 14 days compared to 7 days, with no significant difference between the groups. ALP quantification (Figure 3B) suggested a higher ratio of ALP/total protein in the Ta T70 and Ta T80 groups than in Ta T60 at 7 and 14 days. Qualitative (Figure 3C) and semi-quantitative analysis of ARS staining (Figure 3D) showed a large number of red calcium nodules formed on the surface of all materials at 14 and 21 days, with no statistically significant difference between each other. In addition, we also used RT-PCR to detect the expression of some osteogenic-related genes (ALP, BMP-2, RUNX-2, CXCL-12 and OCN) to compare the osteogenic differentiation of cells on each group of materials at 7 and 14 days, and the results showed that at 7 and 14 days, the expression of ALP, RUNX-2, BMP-2 and CXCL-12 in Ta T70 and Ta T80 groups were higher than those of Ta T60. At 7 days, the difference in OCN between the three groups was not significant, while by 14 days, the expression of Ta T70 and Ta T80 groups was slightly higher than that of Ta T60.

### 3.4 *In vivo* osteogenesis and safety evaluation

To further compare the osseointegration and bone ingrowth ability of three porous tantalum scaffolds with different porosity, we established a rat femoral condylar bone defect repair model. All animals recovered well after the operation, and no adverse reactions such as rejection and infection occurred.

#### 3.4.1 Radiographic results

**FIGURE 4:**
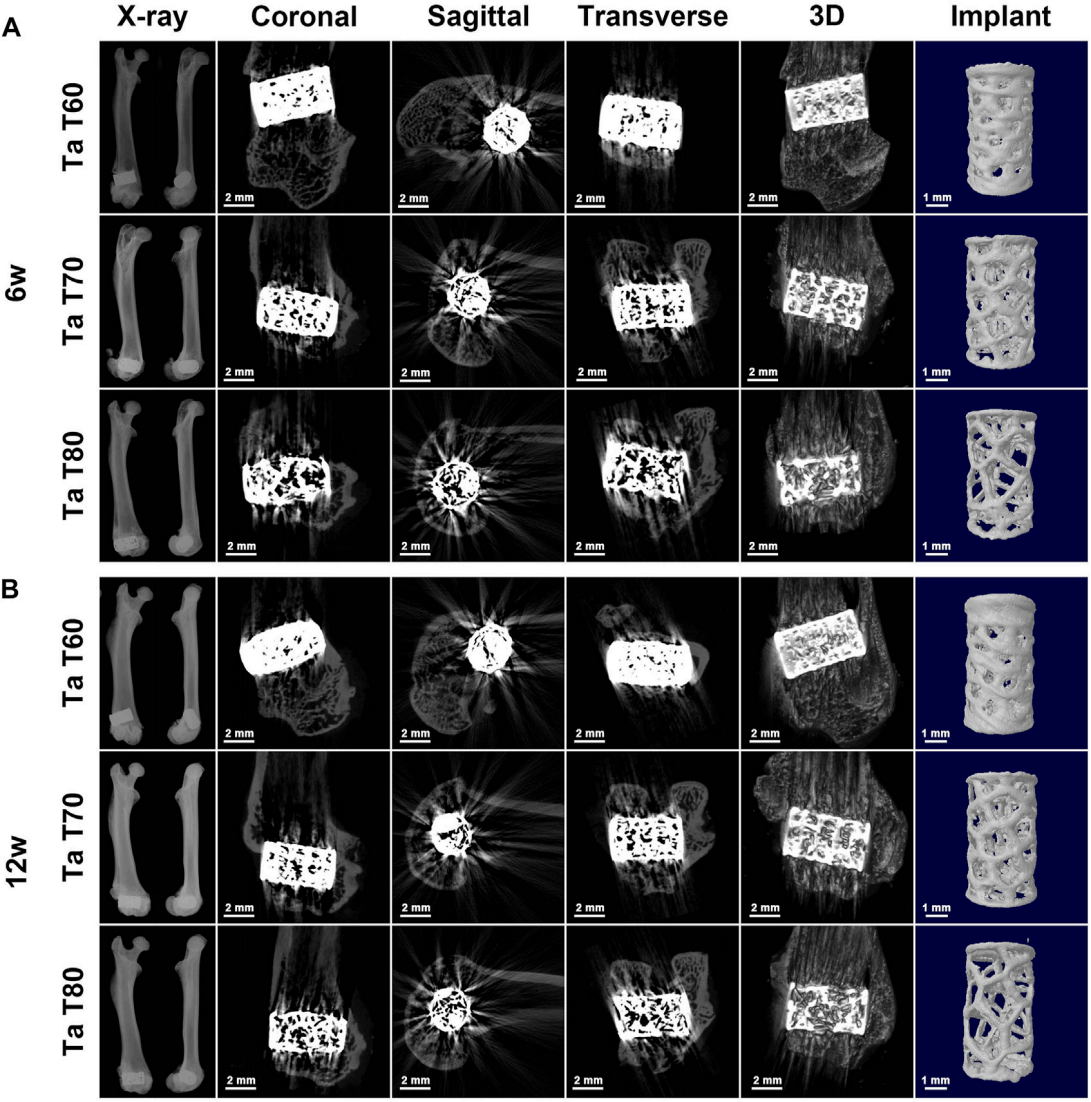
Radiological evaluation of tantalum scaffolds with different porosities implanted in rats. Representative X-ray images and corresponding 2D and 3D construction micro-CT images of the rat femoral condyle as well as the implant at 6w **(A)** and 12w **(B)** after surgery.

The postoperative X-ray results at 6 and 12 weeks (Figure 4) showed that all implants were well positioned and stably integrated with the host bone, with no loosening or dislocation. The porous structure of the implants could be faintly seen in all groups. We also performed micro-CT scans to better present the position of the implants in the femur and the structure of the implants. Figure 4 presents the coronal, sagittal, and transverse 2D images of the three tantalum implants in the femur at 6 and 12 weeks, as well as the reconstructed spatial distribution of the implants in the femur and the 3D morphology of the implants. Because of the high density of tantalum, which can easily absorb X-rays and make them impenetrable, it is not possible to compare the bone ingrowth of the implant well, as many attempts have been made to see more radiographic artifacts around the implant. However, with micro-CT, we can visualize the position of the implant in the femur and identify the shape of the implant.

#### 3.4.2 Results of histological and histometrical analysis

**FIGURE 5:**
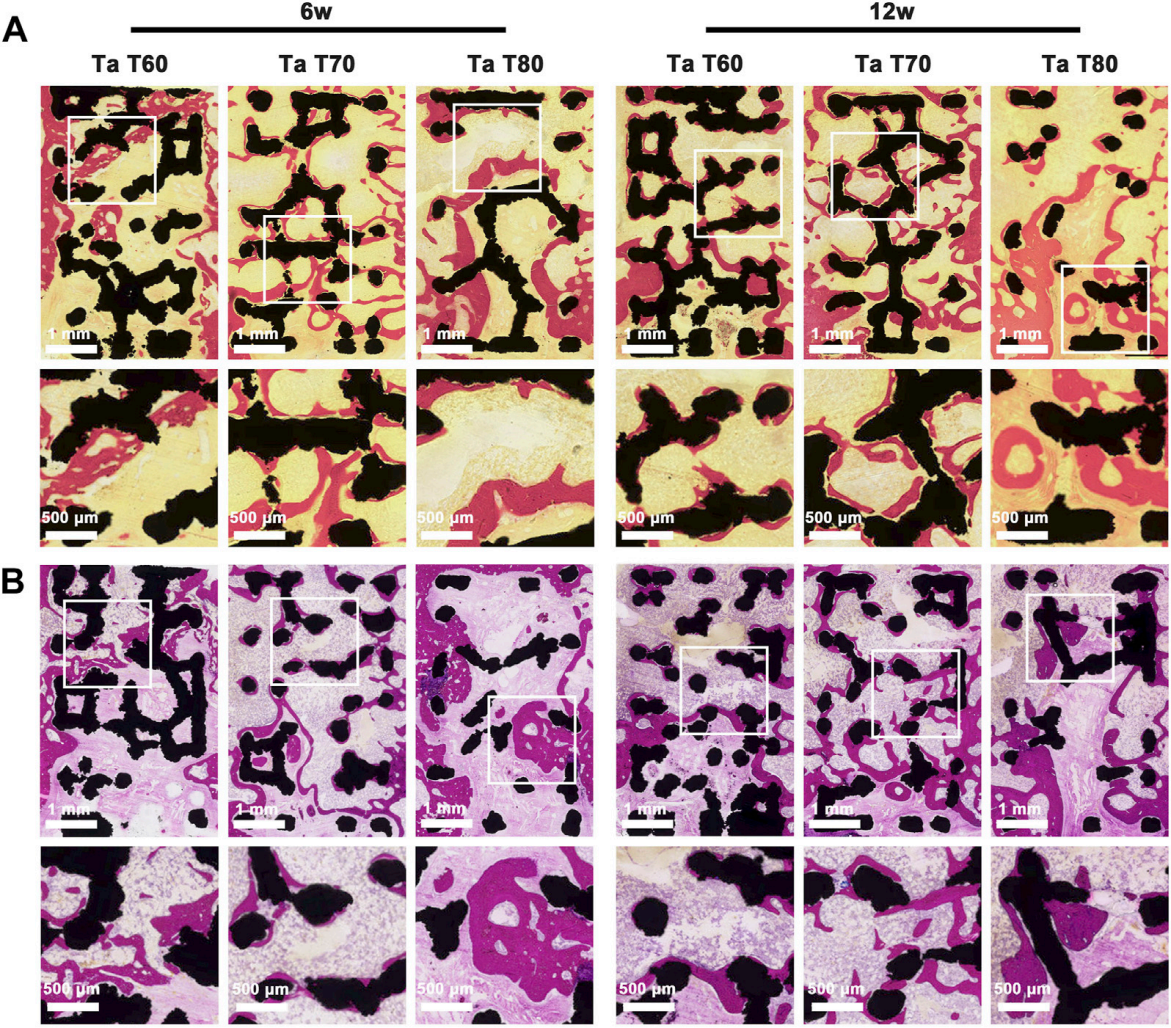
Bone growth *in vivo* evaluated by longitudinal-sectioning of porous tantalum scaffolds at 6w and 12w postoperatively. **(A)** Van Gieson staining and **(B)** methylene blue staining of undecalcified sections.

Next, we performed hard tissue sections of the specimens at 6 and 12 weeks and further evaluated the effect of different porosity on osseointegration and bone ingrowth of porous tantalum scaffolds by VG staining, MB staining, CLSM observation by sequential fluorescence, and SEM. The results of MB staining of the postoperative samples at 6 and 12 weeks are shown in Figure 5A. The global images of the scaffold and the new bone confirmed that the amount of new bone in the three groups of porous tantalum scaffolds with different porosity increased over time, and that the new bone staining purple grew not only at the periphery of the black tantalum scaffold, but also to the interior. The vast majority of them grew against the surface of the tantalum scaffold, in addition to a large amount of granular bone marrow tissue and lamellar collagen fibrous tissue within the material. The large porosity of the scaffold was more favorable for new bone to grow in, and the amount of new bone at 6 weeks was in the order of Ta T80, Ta T70 and Ta T60, but it should be noted that in the Ta T80 group, the bone tissue and the material did not fit closely together and there was more lamellar collagen fiber formation. In contrast, the new bone in Ta T70 was mostly tightly adhered to the material and had more bone marrow tissue. At 12 weeks, the amount of new bone was, in descending order, Ta T70, Ta T80, and Ta T60. Histometric analysis of the ROI area showed that Ta T60, Ta T70, and Ta T80 had 14.3%, 28.6%, and 23.3% of new bone area at 12 weeks, respectively, with BIC indices of 35%, 60%, and 45%, respectively. This result supports that Ta T70 and Ta T80 have better bone ingrowth than Ta T60, and Ta T70 has the best osseointegration ability. In Figure 5B we can see the panoramic and local magnified images of the VG staining of the samples. The red area represents the new bone tissue and the light yellow represents the bone marrow tissue. Similar to MB staining, the RBA at 6 and 12 weeks was in descending order for Ta T80, Ta T70, and Ta T60, but the new bone and material binding of the internal scaffold was not as good in Ta T80 as in Ta T70.

**FIGURE 6:**
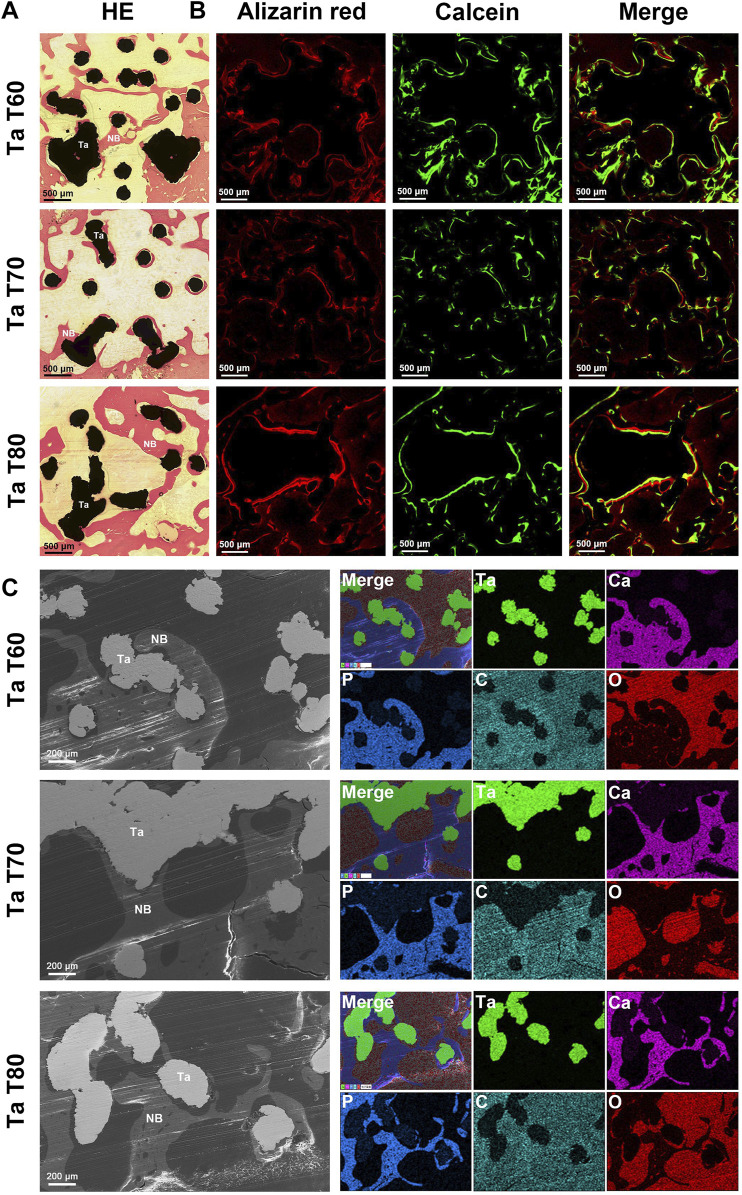
Bone growth *in vivo* evaluated by cross-sectioning of porous tantalum scaffolds at 12w after surgery. **(A)** Representative HE staining results of porous tantalum scaffolds. **(B)** Undecalcified sections of sequential fluorescence staining for bone: red (Alizarin red), green (Calcein) and blue (DAPI). **(C)** SEM micrographs and EDS mapping of bone growth in different porous tantalum scaffolds. Maps of element tantalum, calcium, phosphorus, carbon, and oxygen are in green, pink, blue, cyan, and red, respectively. NB, New bone; Ta, Tantalum.

The results of the cross-section of porous tantalum scaffolds in each group are shown in Figure 6. The VG staining of the transverse section and the staining of the longitudinal section were similar (Figure 6A). New bone grew inward from the perimeter of the scaffold, and the bone growth into Ta T70 and Ta T80 was better than that of Ta T60. The sequential fluorescence staining showed stronger red and green fluorescence in Ta T70 and Ta T80 than in Ta T60 (Figure 6B). SEM and EDS confirmed the presence of new bone and close contact with the scaffold surface (Figure 6C).

#### 3.4.3 *In vivo* biosafety results

**FIGURE 7:**
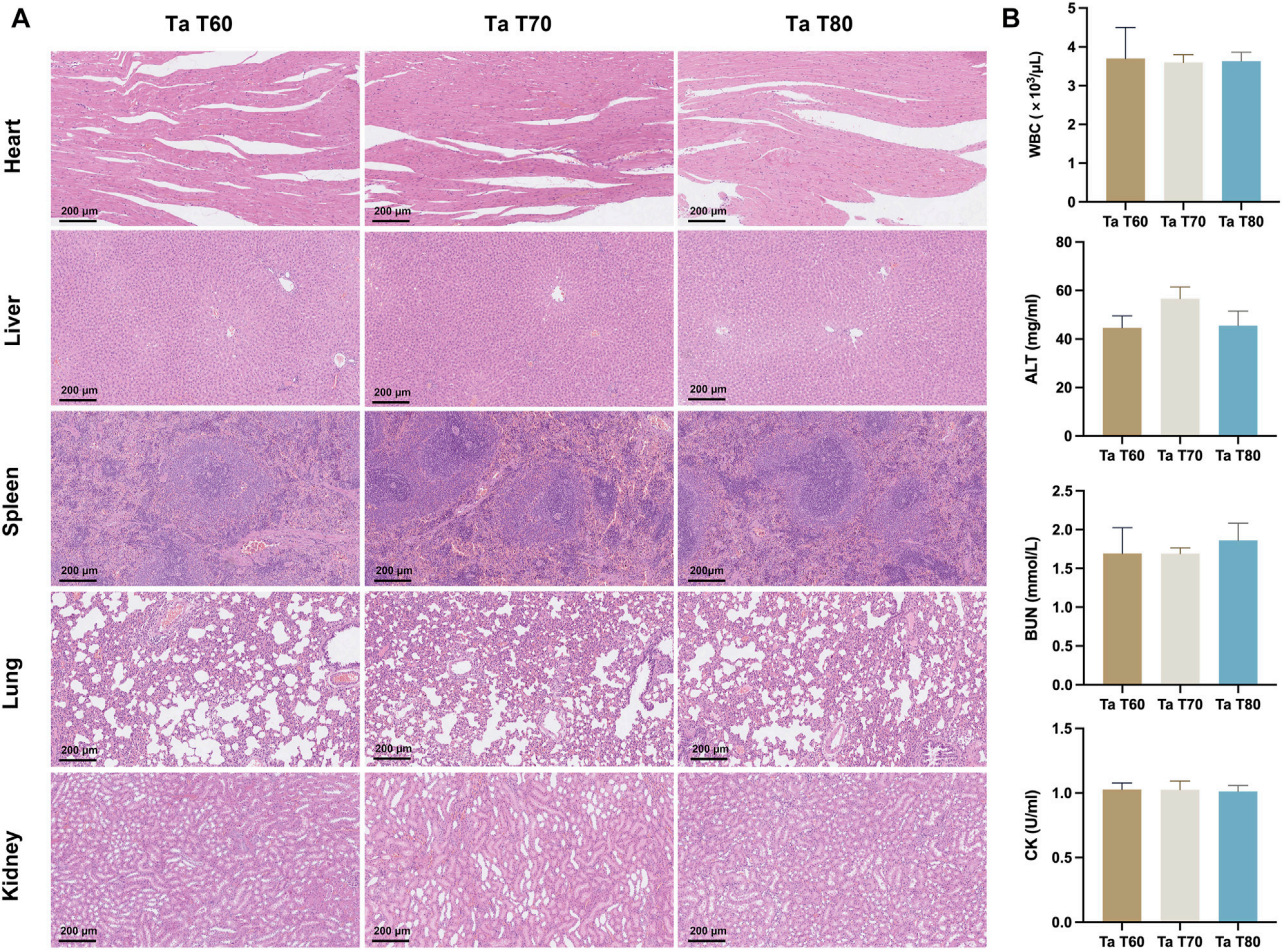
*In vivo* biosafety of porous tantalum scaffolds. **(A)** The HE staining results of rat heart, liver, spleen, lung, and kidney. **(B)** Blood routine and blood biochemical indexes in rats. (n = 3, *p < 0.05; **p < 0.01; ***p < 0.001).

Three groups of rats were euthanized at 12 weeks postoperatively, and heart, liver, spleen, lung and kidney tissues were taken and sections were stained for HE. The results showed that in Figure 7A, no significant abnormal pathological changes were observed in the organ tissue sections of each group. The blood samples from each group were also examined for blood biochemical parameters, and there were no significant statistical differences between the three groups of rats in terms of blood routine, ALT, BUN and CK, all of which were within the normal reference range (Figure 7B). The above results indicate that the porous tantalum scaffold has excellent *in vivo* biosafety.

## 4 Discussion

Bone defects, especially large-sized bone defects of weight-bearing bone, remain one of the most pressing clinical challenges, and porous tantalum stands out for its high porosity, excellent biocompatibility, and suitable elastic modulus. Trabecular Metal prepared by the conventional CVD method is currently widely used in the clinic. However, the disadvantages of the traditional process, such as time-consuming and inefficient, the inability to personalize the material and the lack of precision in its internal structure, have stimulated interest in developing 3D printing technology for the preparation of porous tantalum. The high precision, efficiency and personalization of 3D printing allow us to prepare trabecular porous tantalum scaffolds with different porosity and to study the effects of their mechanical and biological properties. In this study, we successfully prepared three trabecular bone porous tantalum scaffolds with different porosity (60%, 70%, and 80%, respectively) by SLM technology, and *in vitro* studies revealed that although the cell adhesion on the surface of the Ta T60 group was higher than that of the Ta T70 and Ta 80 groups, the proliferation and osteogenic differentiation were inferior to the latter two. The *in vivo* results further confirmed that the Ta T70 and Ta 80 groups showed better bone ingrowth than Ta T60, with Ta T70 having the best osseointegration effect.

In 2015, pure tantalum implants with a high degree of porosity and high interporous interconnectivity were first prepared by Ruben et al. using SLM technology, and a variety of porous tantalum implants with different pore structures have since been prepared (Wauthle et al., 2015; Guo et al., 2019; Wang et al., 2019; Yang et al., 2020). Yang et al. first reported trabecular bone tantalum scaffolds fabricated by AM and compared them with bone trabecular tantalum metal prepared by conventional CVD (Yang et al., 2020). On this basis, porous trabecular tantalum scaffolds with different porosities were prepared. The actual porosity of the three scaffolds was essentially the same as the ideal porosity with an error within 5%, mainly due to the small size of the samples and the different evaluation methods, which was lower than the 99% previously reported (Yang et al., 2020). In addition, except for the differences in porosity and pore size, the strut diameter, inter-pore interconnectivity and micro/nano surface structure were basically the same among the scaffolds, ensuring comparability between groups. Residual powder is a problem that cannot be ignored in metal additive manufacturing processes (Maleki et al., 2021). It has been shown that the angle between the unfused particles and the substrate is less than 90°, which is considered to be beneficial particles that can improve the micro-nano roughness of the material surface and promote the integration with the bone, while if the angle is greater than 90°, it is considered to be harmful particles that can easily fall off from the substrate and cause undesirable performance such as surrounding osteolysis and prosthesis loosening (Pei et al., 2021). The porous tantalum scaffolds in this study were all post-treated by sandblasting, and the surface of the scaffolds was flat, showing a micro/nano-rough structure and essentially free of easily dislodged unfused particles (Figure 1). This post-treatment can better remove residual unfused particles and reduce the impact on 3D printing accuracy and material properties. The porous structure affects the mechanical properties of the material, and the elastic modulus of the trabecular tantalum scaffold fabricated by AM decreases with increasing porosity, but the elastic modulus of the porous tantalum scaffold with all three porosities is within the range of the elastic modulus of human cortical bone and cancellous bone (1.5–3 GPa, 3–30 GPa, and 0.02–2 GPa for porous tantalum scaffolds, human cortical bone, and human cancellous bone, respectively) (Wang et al., 2016; Yang et al., 2020; Wang et al., 2021). Therefore, trabecular tantalum scaffolds are ideal biomaterials for bone filling and bone repair due to their highly porous structure, human bone-matched mechanical properties and rough micro/nano surface structure.

Further, *in vitro* culture of MSCs on scaffolds confirmed the excellent cytocompatibility of trabecular tantalum scaffolds. Both the hemolysis and CCK-8 test confirmed the non-toxicity of the material. Cell adhesion is the first step in the reaction between cell and material, and the physicochemical properties and surface characteristics of the material can have an important impact. Tantalum’s inherent high wettability and surface energy as well as its rough micro/nanosurface structure facilitate protein adsorption and cell adhesion (Huang et al., 2022). SEM showed that MSCs on the surface of each material were fully spread and protruding pseudopodia by 1 day of culture. Live/dead cell staining showed cell growth on the surface of each scaffold after 7 days of culture (Figure 2) and revealed a negative correlation between cell adhesion and the porosity of the material, and, moreover, cells were concentrated on the nodes rather than on the struts. These results are consistent with previous studies (Chen Z. et al., 2020; Pei et al., 2021). Previous studies have shown that the surface area of the material as well as the local curvature have an effect on cell growth. A high surface area facilitates cell adhesion and proliferation, especially under 2D cell culture. In addition, cells prefer to grow on concave (k < 0) and flat surfaces compared to convex surfaces (k > 0) (Zadpoor, 2015). Cells can sense local curvature at the millimeter scale and tend to minimize surface tension at finite volumes, which is thought to be a mechanism that facilitates tissue growth (Rumpler et al., 2008). The struts of three scaffolds in this study had the similar diameter (similar local curvature). The smaller porosity had more scaffold junctions along with a larger surface area, implying relatively more cell adhesion on the surface of Ta T60 scaffold. However, the dead/live cell ratio was slightly higher in the Ta T60 group than in the Ta T70 and Ta T80 groups, and we speculate that the possible reasons for this are excessive cell aggregation or insufficient nutrients delivered due to low porosity. In addition, the relatively high number of residual unfused particles on the surface of Ta T60 may have an effect on the activity of cells.

ALP and calcium deposition were markers of early and late bone formation, respectively. ALP and ARS staining qualitatively and quantitatively showed that porous tantalum scaffolds of all porosities promoted osteogenic differentiation of MSCs, with the Ta T70 and Ta T80 groups outperforming the Ta T60 group. Further PCR assays also supported this result. The porous tantalum scaffolds with high porosity promoted the osteogenic differentiation of MSCs. This phenomenon is consistent with the results of some previous studies. Cheng et al. found that compared with lower porosity scaffolds, scaffolds with high porosities can promote the expression of OCN, OPN, BMP-2, BMP-4 and VGEF (Cheng et al., 2014). Wang et al. found that cellular genes such as ALP, RUNX-2, Col-1 and BMP-2 were more highly expressed in scaffolds with large pore size and porosity (Wang et al., 2022). Besides, in the study of Luo et al., they found that the calcium content of tantalum scaffolds with high pore size and porosity (400–600um/70%, 600–800um/80%) at 21 days of incubation was higher than that of scaffolds with low pore size (100–200um/23%, 200–400/53%), although there was no significant difference in ALP content (Luo et al., 2021). We speculate that the possible reasons for this phenomenon are as follows. First, there would be relatively dense cell distribution, tighter cell contacts, and more extracellular matrix secretion on struts of the scaffold with large porosity after growing the same number of MSCs. This direct cell-to-cell communication induced by cell signaling molecular transmission through gap junctions may significantly enhance osteogenic differentiation of MSCs (Van Bael et al., 2012). Second, large porosity scaffolds have relatively more large pores and more space for cell growth, and the larger distance between struts during attachment and migration may cause cells to produce more stretch to cross the gaps, and more cell stretch facilitates cell differentiation (Kumar et al., 2011). Third, the high permeability due to large porosity could transport more nutrients and oxygen, which may facilitate cell growth. However, some studies have obtained the opposite result. Chen et al. found higher expression of ALP, BMP-2, OPN, OCN and RUNX-2 in Ti6Al4V ELI scaffolds with 60% porosity than in the group with 70% porosity, and attributed this to the low permeability, high inoculation efficiency and high attachment surface area due to the small porosity and small pore size (Chen Z. et al., 2020). In summary, small porosity is more conducive to initial cell adhesion, but the low permeability and easy clogging associated with too small porosity can limit inward cell growth; large porosity is more conducive to cell proliferation and growth, especially inside the scaffold, but too large porosity can affect the mechanical and biological properties of the scaffold (inability to anchor or migrate). Therefore, pore characteristics need to be carefully adjusted to achieve a balance between mechanical, biological and hydrodynamic properties of the scaffold.

We implanted three bionic trabecular tantalum scaffolds into femoral condylar bone defects in rats to further evaluate the osteoconductivity and osseointegration of the material. Radiographic performance at 6 and 12 weeks indicated stable *in vivo* osseointegration of the scaffolds without significant osteolysis or inflammation. Due to the high energy spectrum of tantalum resulting in no X-ray transmission and the large number of metallic artifacts produced in microCT, it was not possible to further assess bone growth within the scaffold by imaging means. Therefore, we chose to use hard tissue sectioning and staining to assess the bonding of the metallic material to the bone. The 3D printed porous tantalum scaffold has superior osteoconduction and osteoincorporation as seen in the hard tissue section results. Once the implant enters the bone defect site, it is in close contact with the surrounding bone, which provides initial stability for successful intraosseous healing of the implant (Davies, 2007). Then, the blood first comes into contact with the implant and a series of biological reactions occur: protein deposition, coagulation, inflammatory response and tissue formation (Kuzyk and Schemitsch, 2011). The surface properties and topology of the implant can have a significant impact on these processes (Gittens et al., 2014; Rupp et al., 2018). The deposition of proteins in turn activates platelets and promotes clotting, i.e., the formation of clots that attach to the implant, and the inflammatory response occurs simultaneously and interacts with platelet activation and clotting (Shiu et al., 2014). The recruitment and migration of osteogenic cells is regulated by fibrin through the clots and possibly by leukocytes and platelets. When osteogenic cells reach the implant surface, they initiate the secretion of bone matrix, preferentially forming a highly mineralized, collagen-free interfacial zone (similar to the cement line of lamellar osteon) (Shah et al., 2019). Differentiated mature osteoblasts continue to secrete collagen outside this zone as well as undergo mineralization to form immature woven bone, which provides secondary stability for implant healing within the host bone while bridging the gap between the implant and the surrounding bone (Davies, 2003). Bone remodeling then occurs in the host bone around the implant and in the immature bone in the interstitial space, resulting in mature lamellar bone and eventual functional healing (Davies, 2003). In this study, the high friction force due to the high friction coefficient of tantalum provided good initial stability, and porous tantalum was more conducive to leukocyte activation and promoted early inflammatory response (Schildhauer et al., 2009). The rough surface topography of the 3D printed tantalum implant provides a larger surface area for protein and platelet adhesion and good osteoconductivity. The porous structure is also more conducive to bone growth and ingrowth. Furthermore, the bone-matched elastic modulus resulting from the porous structure will have a significant impact on later bone remodeling, as there is no significant stress-shielding effect.

3D printing technology has revolutionized the design and preparation process of implants, and the characteristics of precisely tuned porous structures make it possible for us to study the optimal porosity of bone growth. According to RBA and BIC, Ta T70 and Ta T80 have better bone ingrowth effect than Ta T60, among which, Ta T70 has the best osseointegration effect. The large porosity and large pore size of the scaffold can provide more space and attachment area, which facilitates the long entry of bone tissue. In addition, large porosity means more blood and oxygen delivery, and these provide fertile nutrients for bone formation. Several studies have reported the effect of implants with different pore characteristics on bone ingrowth. Taniguchi et al. designed three titanium implants with diamond structures of different pore sizes (300/600/900um) using the SLM technique. The rabbit diaphysis model suggested that the 300um scaffold was less effective in bone ingrowth than the other two groups, and separation experiments suggested that the 600um scaffold had the best bone ingrowth (Taniguchi et al., 2016). Similar results were reported in the study of Ran et al. (Ran et al., 2018). Luo et al. prepared four types of porous tantalum scaffolds with different pore sizes and porosities by SLM, and the *in vivo* results of the rabbit femoral condylar model confirmed that the osseointegration of tantalum scaffolds with large pore sizes (400–600um and 600–800um) was higher than that of scaffolds with low pore sizes (100–200um and 200–400um), with the best osseointegration ability of tantalum scaffolds with 400-600um. They concluded that the effective permeability increases with increasing porosity and pore size, but the effective contact area decreases with increasing porosity and pore size. Moreover, energy dissipation and cell seeding caused by high flow velocity and vortex formation in large pore size scaffolds also have an effect (Luo et al., 2021). Kelly et al. used LPBF to prepare titanium implants with gyroid-sheet architecture of various porosities (0%–90%) and implanted them into the sheep femoral shaft bicortical defect model to systematically investigate the relationship between porosity and implant stiffness, bone ingrowth, and implant-bone mechanical interlocking strength. They found a linear correlation between bone length entry and porosity, but a parabolic relationship between mechanical interlocking strength obtained by osseointegration and porosity, with peaks between 60% and 70%. The interfacial stiffness was inversely linearly related to porosity. The payoff effect of bone ingrowth on osseointegration strength diminished when porosity exceeded 80% (Kelly et al., 2021). The results of these studies are consistent with our results. Of course, there are some studies that do not support this conclusion. Chen et al. reported that the Ti6Al4V ELI porous scaffold prepared by SLM showed the best performance in bone formation (osteogenesis) and bone ingrowth for the scaffold with 500um pore size and 60% porosity compared to the scaffold with 600/700um pore size and 70% porosity (Chen Z. et al., 2020). In the study by Pei et al. (2021) they concluded that 3D printed titanium scaffolds with different pore characteristics (pore size and porosity) had no effect on bone ingrowth outcomes in a rabbit femoral stem cortical defect model and a beagle (beagles) femoral head necrosis model. Instead, implant site had a greater effect on bone ingrowth outcomes. The above results suggest that the design and preparation of materials, the selection of host species and sites, the implementation of experimental methods and the choice of evaluation methods all have an impact on the results, and that uniform and standardized protocols and systems for implant evaluation are needed.

There are still some disadvantages in this experiment. First, we used a rat femoral condylar defect model for the assessment of bone ingrowth effects. Although this is a very common assessment model, the faster bone growth ability of rats may have an impact on the results. Second, we used BIC, a common osseointegration evaluation method in dentistry and orthopedics, to indirectly assess the osseointegration effect, lacking indicators that can directly reflect the bone mechanical interlocking force, such as push-out force and torsion force. These will be further optimized in subsequent studies, such as the use of large animals (sheep, etc.) for modeling and the use of more intuitive mechanical tests for evaluation, for better clinical translation.

## 5 Conclusion

We prepared three porous tantalum implants with different porosity using LPBF. *In vitro* results showed that Ta T60 had more cell adhesion but less cell proliferation and osteogenic differentiation than Ta T70 and Ta T80. *In vivo* bone ingrowth results confirmed that Ta T70 and Ta T80 had better bone ongrowth and bone ingrowth than Ta T60, among which, Ta T70 had the best osseointegration effect. Combined with the *in vivo* and *ex vivo* results, the porous tantalum scaffold with 70% porosity has good osteogenesis, osteoconductivity, osseointegration, biosafety and mechanical properties, and is a very promising 3D printed implant for orthopedics and dentistry, and provides a strong support and reference for the design and optimization of porous tantalum implants afterwards.

## Data Availability

The raw data supporting the conclusions of this article will be made available by the authors, without undue reservation.
