# Supplementary material for: Influence of porosity on osteogenesis, bone growth and osteointegration in trabecular tantalum scaffolds fabricated by additive manufacturing
Source: Front Bioeng Biotechnol. 2023 Jan 27;11:1117954. doi: 10.3389/fbioe.2023.1117954 (PMC9911888; doi:10.3389/fbioe.2023.1117954)
Supplement: Supplementary file 1 [file Image1.pdf]

Supplemental information

Influence of porosity on osteogenesis, bone growth and osteointegration in trabecular tantalum scaffolds fabricated by additive manufacturing

Juyang Jiao[a,1], Qimin Hong[a,1], Dachen Zhang[c,d], Minqi Wang[a], Haozheng Tang[a], Jingzhou Yang[b,c,d,*], Xinhua Qu[a,*], Bing Yue[a,*]

[a] Department of Bone and Joint Surgery, Department of Orthopedics, Renji Hospital Shanghai Jiao Tong University School of Medicine, Shanghai, PR China

[b] School of Mechanical & Automobile Engineering, Qingdao University of Technology, Qingdao, Shandong, PR China

[c] Shenzhen Dazhou Medical Technology Co, Ltd., Shenzhen, Guangdong, PR China

[d] Center of Biomedical Materials 3D Printing, National Engineering Laboratory for Polymer Complex Structure Additive Manufacturing, Baoding, Hebei, PR China

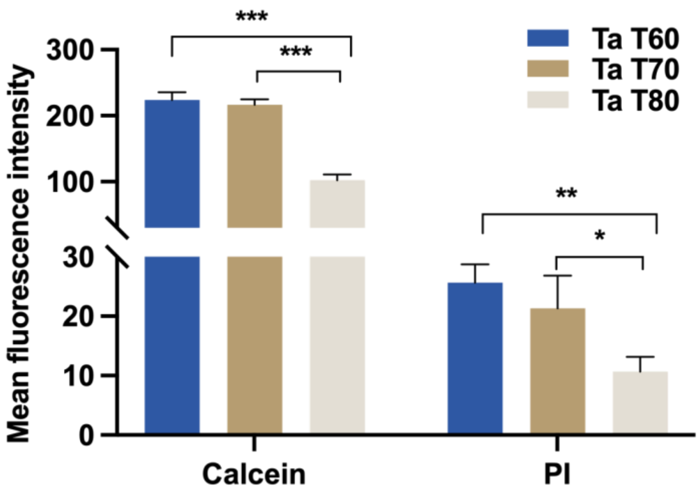

Figure S1. Quantitative analysis of the fluorescence intensity of live/dead cells on different porous tantalum scaffolds. Data represent mean ± SD. (n = 3, *P < 0.05; **P < 0.01; ***P < 0.001).
